# Diabetes Mellitus and Long-Distance Running: A Systematic Review

**DOI:** 10.1186/s40798-026-01084-z

**Published:** 2026-08-03

**Authors:** Lorin Braschler, Mabliny Thuany, Thomas Züger, Pantelis T. Nikolaidis, Katja Weiss, Thomas Rosemann, Beat Knechtle

**Affiliations:** 1 Faculty of Medicine, University of Bern, Bern, Switzerland; 2 Department of Sports, States University of Pará, Belém, PA Brazil; 3 Department of Endocrinology and Metabolic Diseases, Kantonsspital Olten, Olten, Switzerland; 4 Department of Diabetes, Endocrinology, Nutritional Medicine and Metabolism, Inselspital, Bern University Hospital, University of Bern, Bern, Switzerland; 5 School of Health and Caring Sciences, University of West Attica, Athens, Greece; 6 Institute of Primary Care, University of Zurich, Zurich, Switzerland; 7 Medbase St. Gallen Am Vadianplatz, Vadianstrasse 26, 9001 St. Gallen, Switzerland

**Keywords:** Endurance athletes, Glycemic control, Continuous glucose monitoring, Insulin management, Hypoglycemia risk

## Abstract

**Background:**

Athletes with diabetes increasingly participate in endurance running, benefiting from physiological adaptations and technological advances like continuous glucose monitoring (CGM) for glycemic control. So far, no comprehensive review summarizing the current knowledge has been performed.

**Objective:**

To identify the prevalence of long-distance runners with diabetes, their performance, methods used for blood glucose monitoring and glycemic control.

**Methods:**

PubMed, Medline Ovid, Scopus, SPORTDiscus, Cochrane databases, CINAHL, and Web of Science were systematically searched until April 2024 using key terms related to long-distance running and diabetes. An updated search applying the identical strategy was conducted on May 3rd, 2026, to capture subsequently published literature. This study included original research articles, case reports, and case studies published in English or German in peer-reviewed journals, utilizing both quantitative and qualitative methodologies. Eligibility criteria encompassed runners with a confirmed diagnosis of diabetes mellitus, irrespective of type, who participated in endurance events of at least half-marathon distance, including half-marathons, marathons, ultra-marathons, and other ultra-endurance competitions. Patients with prediabetic state, gestational diabetes, or after pancreatic islet-cell transplantation were excluded. For quality assessment, we used the Joanna Briggs Institute of Analytical Cross-Sectional Studies or the Joanna Briggs Institute of Analytical Case Report critical appraisal tool.

**Results:**

A total of 656 studies were identified, with 22 meeting the inclusion criteria, comprising 99 runners with diabetes (99.0% Type 1, 1.0% Type 2 diabetes mellitus). Among them, 50.0% used CGM, 27.2% had an insulin pump, 63.6% administered insulin via multiple daily injections, and the method of insulin application was not specified in 7.0% of runners. The weighted mean HbA_1c_ was 7.4% (95% CI 6.9–8.1). Time-in-range varied by race distance: 40–100% for half-marathoners, 51.6% for marathoners, and 47–73% for ultra-marathoners. No cases of symptomatic hypoglycemia were observed during the races. However, asymptomatic late-onset hypoglycemia (occurring 6–15 h post-exercise) was reported in 27 runners. The most common insulin adjustment strategy before competitive races was a 50–80% reduction of basal insulin.

**Conclusion:**

Despite limited research, evidence suggests that with proper preparation and multidisciplinary support, athletes with diabetes can safely participate in endurance events, including ultra-marathons, while maintaining stable glycemic control.

*Trial Registration*: PROSPERO—CRD42024539281.

## Key Points

- Long-distance runners with diabetes maintained moderate hyperglycemia to prevent hypoglycemia during races.
- A relatively low incidence of late-onset hypoglycemia was reported.
- The most common strategy to prevent exercise-induced hypoglycemia was a basal insulin reduction of 50–80%.
- CGM use was the most common form of glucose monitoring
- Biomarkers of stress-related hormonal changes responded similarly compared to healthy controls.

## Introduction

Long-distance running is practiced all over the world by an increasing number of individuals, independently of sex and age, who benefit from the physiological and psychological adaptations to this type of exercise [1, 2]. In this context, it is not surprising that also individuals with non-communicable diseases such as diabetes are interested in running for either only training purposes or participating in a competitive sport event such as a marathon [3, 4]. Until a few decades ago, there was limited evidence supporting the beneficial role of endurance exercise such as running for humans with diabetes [5, 6]. Individuals with diabetes often struggle to maintain glycemic targets during running, which can pose a significant challenge. This difficulty may lead to avoidance of sports due to the fear of hypoglycemia [7]. Nevertheless, the advancement of science and technology nowadays allows the participation of these patients in running, and the question is not whether they should run, but how they should run safely [7, 8]. Thus, it is not surprising to observe unique achievements by runners with diabetes such as finishing 1000 marathon races [9] and 48 24-h ultra-marathons [10].

The existing literature covers a wide range of running events ranging from 10 km to half-marathon [7, 11, 12], marathon [3, 13, 14], ultra-marathons (e.g., time-limited formats such as 24-h [15] and 6 days [10], mountain race [8], and triathlons of different distances [16]. In addition, studies have examined acute responses to running for training purposes such as 2-h running on the road [3] or on a treadmill [17]. A major concern of runners with diabetes has been the prevention of hypoglycemia (i.e., blood glucose < 3.9 mmol/l [70 mg/dl]) while staying in euglycemia (3.9–11.1 mmol/l [70–200 mg/dl]). Prevention strategies include carbohydrate intake, real-time continuous glucose monitoring (CGM) and insulin dose adjustments [3, 15]. Accordingly, it has been suggested that muscle, liver and glycogen metabolism can be normal in runners with diabetes given an optimal glucose management, adjustments to insulin dose and nutrition [10].

The abovementioned studies have improved the understanding of the characteristics of runners with diabetes, as well as their physiological responses and adaptations to running. However, no review has ever been conducted about the acute effect and chronic adaptations of individuals with diabetes to long-distance running so far. Such a review would have practical applications for professionals working with runners with diabetes in the context of optimal guidance. Furthermore, considering the existence of many case studies [7, 9, 10, 14] and original research studies with small sample sizes [11, 12, 16, 17], it is important to summarize and evaluate their findings. To our knowledge, there is no systematic review ever conducted investigating long-distance runners with diabetes. Therefore, the aim of the present study was to critically review the existing literature about the prevalence of long-distance runners with diabetes, their performance, training characteristics, and methods used for blood glucose monitoring. As additional outcomes, we aimed to analyze the effect of long-distance running on glycemic control and cardiovascular risk profile of long-distance runners with diabetes, prevalence of CGM use in runners with diabetes and its implications and advantages for runners without diabetes using blood glucose monitoring systems during competitions.

## Methods

### Protocol and Registration

This systematic review was performed according to the 2020 Preferred Reporting Items for Systematic Reviews and Meta-Analyses (PRISMA) statement [18]. The corresponding protocol was registered at the International Prospective Register of Systematic Reviews (PROSPERO—CRD42024539281).

### Database and Search Strategy

A systematic literature search was performed and the following databases were searched for appropriate literature: PubMed, Medline Ovid, Scopus, SPORTDiscus, Cochrane databases, CINAHL, and Web of Science. The search strategy was established by the first author (L.B.) in conjunction with this review’s co-authors (M.T., B.K.). Medical subject headings (MeSH) terms were combined with free-text search. The search comprised two sets of keywords which in turn were coupled using the Boolean operator AND. The first one covered the different disciplines in long-distance running. All key terms were combined with the OR operator (“marathon*”, “marathon run*”, “half-marathon*”, “half-marathon run*”, “ultramarathon*”, “ultra-marathon*”, “ultra-marathon run*”, “ultramarathon run*”, “triathlon*”, “ultra-triathlon*”). The second set covered different key terms for diabetes mellitus, which were combined with the Boolean operator OR ("adult-onset diabetes mellitus", "autoimmune diabetes*", "brittle diabetes mellitus*", "cardiometabolic syndrome*", "cardiometabolic syndromes", "cgm device*", "cgm device*", "CGM",, "closed loop system*", "closed-loop system*", "closed-loop-system*", "compensatory hyperinsulinemia", "continuous glucose monitoring device*", "continuous glucose monitoring*", "diabetes mellitus*", "dysmetabolic syndrome x", "endogenous hyperinsulinism", "exogenous hyperinsulinism", "fasting hypoglycemia", "hyperglycemi*", "hyperinsulinemia", "hyperinsulinism", "hypoglycemi*", "IDDM", "insulin dependent diabetes mellitus 1", "insulin pump*", "insulin resistance syndrome x", "insulin-dependent diabetes mellitus", "insulin-pump*", "juvenile onset diabetes", "juvenile-onset diabetes mellitus", "ketosis-prone diabetes mellitus", "ketosis-resistant diabetes mellitus", "LADA", "latent autoimmune diabetes in adults", "latent autoimmune diabetes of adults", "maturity onset diabetes mellitus", "maturity onset diabetes", "metabolic cardiovascular syndrome*", "metabolic syndrome x", "metabolic syndrome*", "metabolic syndromes", "metabolic x syndrome*", "MODY", "niddm", "non-insulin-dependent diabetes mellitus", "postabsorptive hypoglycemi*", "postprandial hypoglycemi*", "reactive hypoglycemi*", "reaven syndrome x", "stable diabetes mellitus", "sudden-onset diabetes mellitus", "T1DM",, "T2DM",, "T3DM",, "type 1 diabetes", "type 2 diabetes"). The full literature search was completed in April 2024 and updated on 3rd May 2026. All retrieved articles were uploaded into Rayyan, an artificial intelligence (AI) supported semi-automated tool specifically designed to facilitate and assist in the initial screening process of abstracts in systematic reviews [19]. All found duplicates were checked and removed manually with corresponding suggestions from Rayyan.

### Study Inclusion and Exclusion Criteria

This systematic review included original articles, case reports, and case studies published in English or German that investigated athletes with a confirmed diagnosis of diabetes mellitus (e.g., Type 1, Type 2, and maturity-onset diabetes of the young, etc.) according to the American Diabetes Association criteria defined as having at least one of the following: fasting plasma glucose ≥ 7.0 mmol/l (126 mg/dL) after at least 8 h without caloric intake, 2-h plasma glucose ≥ 11.1 mmol/l (200 mg/dL) during a 75 g oral glucose tolerance test, glycated hemoglobin HbA_1c_ ≥ 6.5% (48 mmol/mol), or random plasma glucose ≥ 11.1 mmol/l (200 mg/dL) accompanied by classic symptoms of hyperglycemia or a hyperglycemic crisis. Eligible participants competed in endurance events of at least half-marathon (21.0975 km) [20], marathon (42.195 km) [21], ultra-marathon (> 42.195 km, > 6-h) [22], or other ultra-endurance events such as ultra-triathlons and IRONMAN^®^ triathlons with at least half-marathon distance. Athletes of all sexes, ages, and performance levels (professional, recreational, amateur) participating in road, off-road (including trail running), or treadmill events were included.

Exclusion criteria encompassed athletes with prediabetes, defined as HbA_1c_ 5.7–6.4%, fasting glucose 5.6–6.9 mmol/l (100–125 mg/dl), or 7.8–11.0 mmol/l (140–199 mg/dl) 2 h after oral glucose tolerance test according to the American Diabetes Association [23]. Furthermore, athletes with gestational diabetes, and athletes with diabetes after pancreatic islet-cell transplantation were not considered for inclusion. Additionally, animal, or in vitro studies and not having access to full-text were excluded. All studies published until April 2024 were considered for inclusion. An updated literature search was performed on May 3rd, 2026, applying the identical search strategy used in April 2024 to capture all subsequently published literature. The PRISMA flow-chart was updated accordingly, and any additional articles identified as potentially eligible were assessed for inclusion.

### Study Selection

After duplicate removal, the titles and abstracts of all articles were screened by two reviewers (L.B., M.T.) independently and blinded to each other’s assessment in Rayyan without the use of AI support. The potentially relevant articles selected in title and abstract screening were further examined in full text by two reviewers (L.B., M.T.) independently and in a blinded manner. For inclusion in the final review, selected articles were approved by two reviewers (L.B., M.T.). In cases of disagreement, these were resolved by discussion with a third independent reviewer (B.K.) blinded to the decision of the other two parties.

### Data Extraction and Quality Assessment

Data extraction from all included studies was conducted by one reviewer (L.B.), comprising: (1) authors; (2) date of publication; (3) study design; (4) sample characteristics; (5) diabetes characteristics (i.e., type of diabetes, mean HbA_1c_, etc.); (6) running history and training; (7) race characteristics and performance; (8) glucose monitoring during the race including time-in-range (TIR), time-below-range (TBR), time-above-range (TAR), hypoglycemic, and hyperglycemic events, respectively; (9) key parameters assessed; and (10) main results.

We classified runners based on their weekly training history and assigned them a performance level (i.e., recreational, amateur, or elite). Recreational runners were defined as finishing ≤ 3 training sessions or ≤ 35 km total training mileage per week [24]. On the other hand, amateur runners were defined as completing > 3 training sessions or > 35 km total mileage per week [24], whereas elite athletes completed ≥ 100 km per week. Hypoglycemia, hyperglycemia and TIR were defined (see Table 1) in accordance with the consensus definition by the American Association of Clinical Endocrinologists, the American Association of Diabetes Educators, the American Diabetes Association, and others [25].

**Table 1:** Definitions of hypoglycemia, hyperglycemia and TIR according to the consensus definitions of the American Association of Clinical Endocrinologists, and others (definitions adopted from [25])

Glycemic parameter	Definition
Hypoglycemia	Blood glucose < 3.9 mmol/l (70 mg/dl)
Hyperglycemia	Blood glucose > 10 mmol/l (180 mg/dl)
TIR	Proportion of glucose readings within 3.9–10.0 mmol/l (70–180 mg/dl) per unit of time

*TIR* time-in-range

The quality of all included studies was assessed individually and blinded to each other’s judgment by two reviewers (L.B., M.T.) using either the Joanna Briggs Institute of Analytical Cross-Sectional Studies or the Joanna Briggs Institute of Analytical Case Report critical appraisal tool (see Table 2) [26]. Disagreements were resolved by consensus of the two reviewers. The checklist for cross-sectional studies included eight criteria: (1) definition of included samples; (2) detailed description of study subjects and setting; (3) valid and reliable measurement of exposure; (4) use of objective and standard criteria for the condition; (5) identification of confounders; (6) strategies applied to deal with confounders; (7) reliable and valid measurement of outcomes; (8) appropriate statistical analysis. Similarly, the checklist for case reports included eight criteria: clear description of (1) demographic characteristics; (2) patient’s history and timeline; (3) clinical condition; (4) diagnostic tests, assessment methods and results; (5) intervention or treatment procedures; (6) post-interventional clinical condition; (7) adverse or unanticipated events; (8) provided takeaway lessons. Each criterion was scored as being “met = yes” or “not met = no” or “unclear”. Each criterion that was scored as “met = yes” scored one point with the maximum attainable score of 8. The results of this assessment were used to assign an a priori quality rating to each study (0–2 points = very low; 3–4 points = low; 5–6 points = moderate; 7–8 points = high quality). All extracted numerical data are given as mean ± standard deviation unless stated otherwise.

**Table 2:**
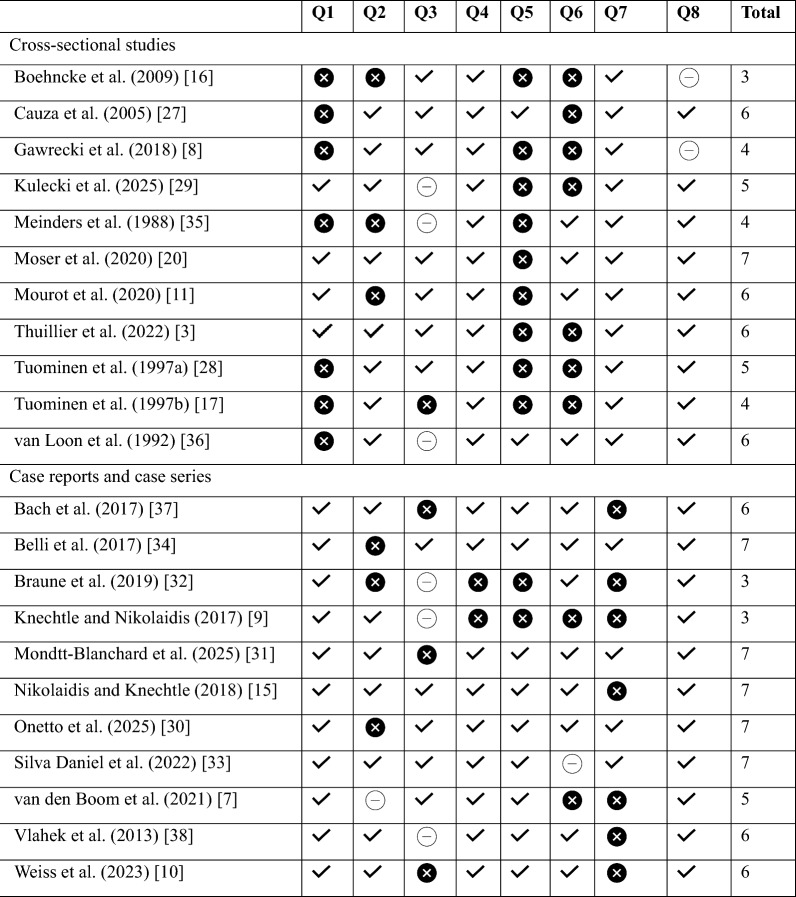
Systematic quality check of included studies

Cross-sectional studies: Q1 Were the criteria for inclusion in the sample clearly defined?; Q2 Were the study subjects and the setting described in detail?; Q3 Was the exposure measured validly and reliably?; Q4 Were objective, standard criteria used for measurement of the condition?; Q5 Were confounding factors identified?; Q6 Were strategies to deal with confounding factors stated?; Q7 Were the outcomes measured validly and reliably?; Q8 Was appropriate statistical analysis used? Case reports and series: Q1 Were patient’s demographic characteristics clearly described?; Q2 Was the patient’s history clearly described and presented as a timeline?; Q3 Was the current clinical condition of the patient on presentation clearly described?; Q4 Were diagnostic tests or assessment methods and the results clearly described?; Q5 Was the intervention(s) or treatment procedure(s) clearly described?; Q6 Was the post-intervention clinical condition clearly described?; Q7 Were adverse events (harms) or unanticipated events identified and described?; Q8 Does the case report provide takeaway lessons? Yes ( ); No ( ); Unclear ( ). The results of this assessment were used to assign an a priori quality rating to each study (0–2 points = very low; 3–4 points = low; 5–6 points = moderate; 7–8 points = high quality)

### Statistical Analysis

Weighted mean values for continuous outcomes were calculated using a random-effects model analogous to meta-analytic procedures, with subgroup analyses performed by publication date. Heterogeneity across studies was assessed using the Q-test, and statistical significance was set at *p* < 0.05. All analyses were conducted using Stata/MP18.0 (StataCorp, College Station, TX, USA). All extracted numerical data are given as mean ± standard deviation unless stated otherwise.

## Results

### Literature Search

**Fig. 1:**
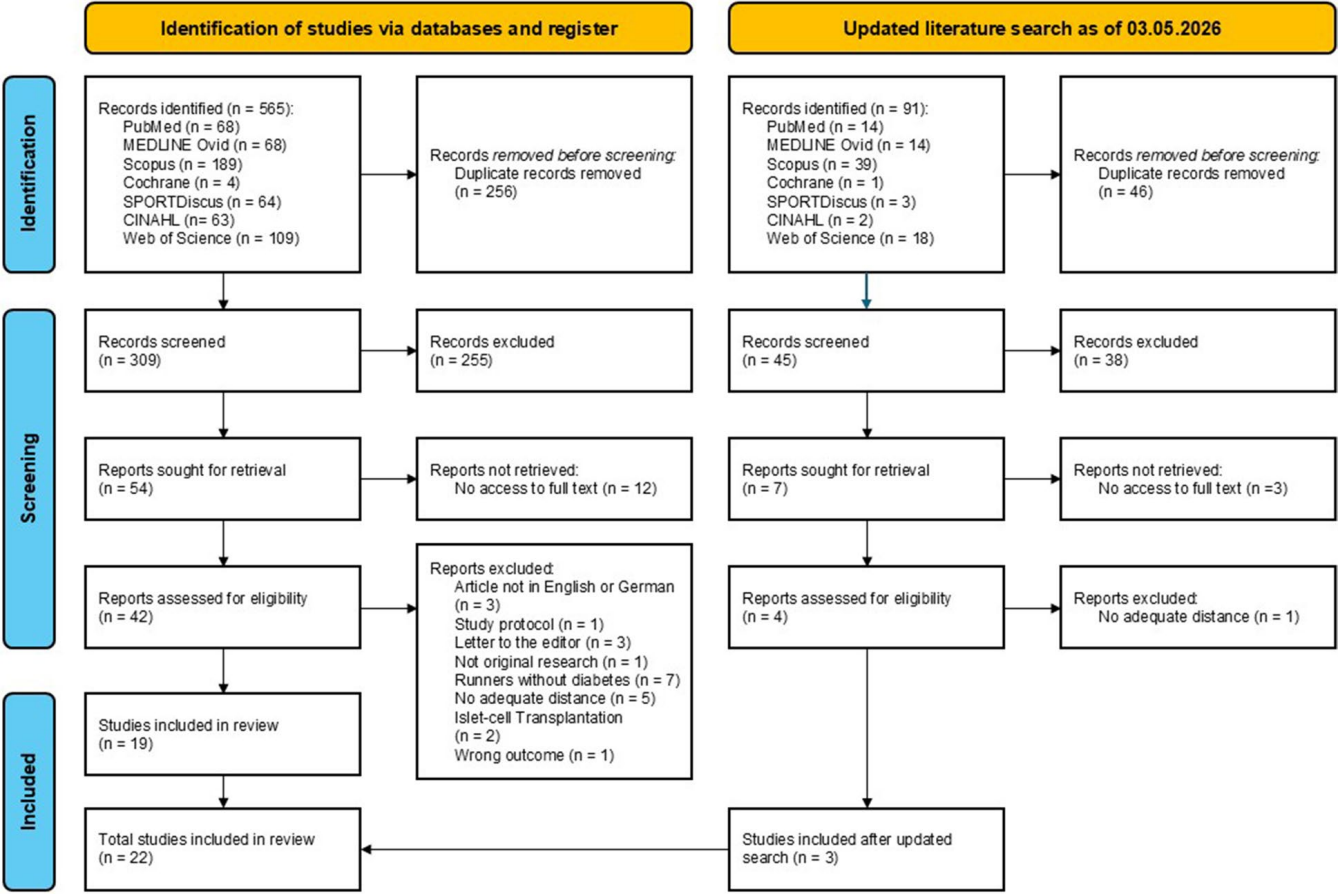
PRISMA flow diagram of the study screening and selection process

In total, our search strategy identified 565 studies. After duplicate removal, 309 studies were included for title and abstract screening, of which 54 were considered relevant for our review and screened in full-text. Overall, 19 studies met the inclusion criteria and were included in this review. The updated literature search identified an additional 91 studies, 45 were evaluated in the title and abstract screening, 7 were assessed as full-text and 3 were deemed eligible for the systematic review. The full screening process (PRISMA flow-chart) is depicted in Fig. 1.

### Quality Appraisal

The systematic quality check of included studies is shown in Table 2. The agreement rate between the two reviewing authors was 92.8% overall, 93.8% for cross-sectional studies, and 91.7% for case reports and series. For the cross-sectional studies, all of them were classified as “high quality” or “moderate”, while for the case reports, five were considered “moderate” and six as “low”. In these studies, study subjects and setting were accurately described (Q2), conditions (Q4) and outcomes (Q7) were measured in an objective and standardized way; however, only a third of the studies clearly specified criteria for inclusion in the study sample (Q1), and most of them failed to provide information about the confounders and strategies applied to deal with confounders. Regarding case reports and case series, most studies clearly described patients’ demographic characteristics (Q1) and medical history (Q2), as well as post-interventional clinical condition (Q6). However, some studies failed to mention actual clinical data (Q3) regarding patients’ condition (e.g., latest HbA_1c_ values) and intervention or treatment procedures.

### Study and Sample Characteristics

Tables 3 and 4 display the key aspects of the included studies. Data were acquired from 161 participants, of whom 140 (87.0%) were men, and 21 (13.0%) were women. The publication date ranged from 1988 to 2026, with most studies being published after 2017 (68.2%). Eleven studies (50.0%) were designed as prospective studies, and 11 studies (50.0%) were case reports. Three studies (13.6%) were written in German (consisting of one prospective study and two case reports), whereas 16 studies (86.4%) were published in English. In regard to running distance, 8 studies (36.4%) included marathon [3, 9, 17, 27–31], 4 studies (18.2%) half-marathon [7, 11, 20, 32], 5 studies (22.7%) ultra-marathon (82-217 km and 24-h) [8, 10, 15, 33, 34], and 5 studies (22.7%) included other distances (3-h runs, IRONMAN^®^, Double Iron ultra-triathlon) [16, 35–38]. Given the running history of all participants, 72 (44.7%) were classified as recreational, 82 (50.9%) as amateur, and 6 (3.7%) were regarded as elite runners. Out of all participants, 43 (26.7%) had previously partaken in a marathon, 18 (11.2%) finished a half-marathon, 22 (13.7%) had no previous marathon experience, and the running history was not stated in 78 (48.4%) of runners.

**Table 3:** Summary of study and population characteristics, methods, and key findings of included cross-sectional studies

Study	Design	Sample characteristics	Diabetes characteristics	Running history	Race characteristics and performance	Glucose monitoring during the race	Key parameters assessed	Main results
Boehncke et al. (2009) [16]	Prospective, observational study	*N* = 10 Female = 0 Age (years) range 32–61	Type 1 (*N* = 10) Duration (years) range 2–35 CSII (*N* = 3) MDI (*N* = 7)	Recreational Training volume (h/week) marathon group 20 ± 0, 16 km group 16 ± 0	Ironman Germany^®^ (3.86 km swimming, 180.2 km cycling, 42.195 km running) Finishing time (h) range 10.8–14.9 Carbohydrate intake (g/h) range 24–60	Manual (*N* = 10) Hyperglycemic^a^ events (*N* = 5)	Metabolic and hormonal changes	Successful completion of Ironman Germany^®^ of runners with diabetes with comparable finishing times to healthy age-matched participants Basal insulin dose reduction by up to 50% on race day Similar hormonal changes in adrenal hormones, testosterone, TSH, fT3, fT4 between participants with and without diabetes
Cauza et al. (2005) [27]	Prospective, observational study	*N* = 6 Female = 4 Age (years) male 37 ± 11, female 39 ± 2.5 BMI (kg/m^2^) 23.2 ± 1.1	Type 1 (*N* = 5) Type 2 (*N* = 1) HbA_1c_ (%) 7.5 ± 0.8 CSII (*N* = 2) MDI (*N* = 4)	Recreational Training volume (h/week) marathon group 20 ± 0, 16 km group 16 ± 0	Vienna City Marathon (42.195 km) and 15.8 km run Finishing time (min) marathon 257.3 ± 14.6, 16 km 134.0 ± 19.0	CGM (*N* = 6) Hypoglycemic events^b^ (*N* = 2) Hyperglycemic events^a^ (*N* = 1)	Glycemic dynamics during the race and the night after	Asymptomatic mild hypoglycemic episodes < 3.3 mmol/l (60 mg/dl) during the race in two participants No prolonged hyperglycemic episodes Prolonged mild hypoglycemia in the night after the race in one participant One episode of hyperglycemia 19.4 mmol/l (350 mg/dl) the night after the race Long-term glycemic control improved with endurance training
Gawrecki et al. (2018) [8]	Prospective, observational study	*N* = 4 Female = 0 Age (years) 35.5 ± 8.1 BMI (kg/m^2^) 24.4 ± 0.4	Type 1 (*N* = 4) HbA_1c_ (%) 6.7 ± 0.5 Duration (years) 15.0 ± 11.7 TDI (IU/kg) 44.2 ± 11.9 CSII (*N* = 4)	Amateur Previous marathon participation (*N* = 4)	Mountain ultra-marathon (82 km, climbs 3,235 m, descents 3,055 m) Finishing time (h) 14.6 ± 1.2 Carbohydrate intake (g/h) 37.4 ± 7.4	CGM (*N* = 4) Hypoglycemic events^b^ (*N* = 1) Hyperglycemic events^a^ (*N* = 5)	Glucose, ketone and lactate kinetics	No metabolic complications No improvement or deterioration of glycemic control the following 24-h post-race Ketone levels did not exceed 1.5 mmol/l Elevation in lactate levels comparable to healthy controls
Kulecki et al. (2025) [29]	Prospective, observational study	*N* = 5 Female = 0 Age (years) median 44 (IQR 34–48)	Type 1 (*N* = 5) HbA_1c_ (%) 5.8 (IQR 5.6–6.9) Duration (years) median 10 (IQR 6–14) MDI (*N* = 5)	Amateur Previous marathon participation (*N* = 3)	Poznan Marathon (42.195 km) Finishing time (min) Mean 242.9 ± 43.2	CGM (*N* = 5) Hypoglycemic events^b^ (*N* = 3) Hyperglycemic events^a^ (*N* = 1)	Glycemic control and differences between self-monitored blood glucose measurements and CGM	Safe completion of marathon CGM accuracy was reduced during exercise Pre-exercise glycemia was a key predictor of in-race hypoglycemia
Meinders et al. (1988) [35]	Prospective, controlled observational study	*N* = 10 Female = 0 Age (years) T1DM 35.4 (range 30–42), controls 36.4 (range 24–52) BMI (kg/m^2^) T1DM 22 (range 21.5–23.2), control 23.6 (21.9–27.2)	Type 1 (*N* = 5) HbA_1c_ (%) T1DM 10.6 (range 9.0–11.8), control 7.3 (range 7.0–7.8) Duration (years) range 0.25–5 TDI (IU) range 12–48 MDI (*N* = 5)	Recreational	3-h run at participants usual marathon pace	Manual (*N* = 5)	Metabolic and hormonal changes in T1DM without morning insulin dose	Significant decrease in blood glucose concentrations during the 1.5 h after the run in runners with diabetes Significant increase in serum lactate and pyruvate concentrations in runners with diabetes Significantly higher increase in β-hydroxybutyrate concentrations in runners with diabetes compared to healthy controls during the hour after the run
Moser et al. (2020) [20]	Prospective, single center, controlled observational study	*N* = 24 Female = 9 Age (years) T1DM 41 ± 12, controls 38 ± 6 BMI (kg/m^2^) T1DM 25.5 ± 3.0, controls 22.9 ± 2.8	Type 1 (*N* = 12) HbA_1c_ (%) T1DM 7.5 ± 1.5, controls 4.6 ± 0.3 Duration (years) 12 ± 10 TDI (IU) 37 ± 14 CSII (*N* = 7) MDI (*N* = 5)	Recreational	Half-marathon (21.2 km, T1DM *N* = 5, control *N* = 6) and 10km (T1DM *N* = 7, control *N* = 6) Total competition time (min) T1DM 85 ± 29, controls 89 ± 37 Carbohydrate intake (g) T1DM 27 (IQR 0–47), controls 17 (IQR 0.28)	CGM (*N* = 12) TIR^a^ (%) T1DM median 40 (IQR 0–60), controls median 100 (IQR 100–100) TBR (%) T1DM median 0 (IQR 0–0), controls median 0 (IQR 0–0) TAR (%) T1DM median 20 (IQR 2–29) for TAR 1 (> 10.0–13.9mmol/l) and median 9 (IQR 0–55) for TAR 2 (> 13.9 mmol/l) Controls median 0 (IQR 0–0) for TAR 1 and 2	Glycemic dynamics and macronutrient intake	Significantly higher glycemic variability in runners with diabetes compared to healthy controls during the run Healthy controls did not maintain a TIR^a^ of 100% throughout the entire observation period, while TIR was 100% during the race, they still showed minor TBR episodes both pre- and post-competition Significant reduction in basal insulin dose in runners with diabetes during the competition No hypoglycemic events during the competition in neither group No events of late-onset hypoglycemia
Mourot et al. (2020) [11]	Cross-sectional study	*N* = 18 Female = 2 Age (years) T1DM 39.0 ± 11.1, controls 42.4 ± 5.8 BMI (kg/m^2^) T1DM 23.1 ± 2.4, controls 23.3 ± 1.9	Type 1 (*N* = 9)	Recreational Training volume (h/week) 3.7 Previous half-marathon (*N* = 18)	Half-marathon (21.2 km) Finishing time (min) T1DM 111.0 ± 18.7, controls 104.0 ± 13.2	Not specified	Cardiovascular and neuro-hormonal response to acute exercise	No significant differences between runners with diabetes and healthy athletes regarding cardiovascular and autonomic variables Post-exercise hypotension one-hour post-exercise with increased sympathetic activity, decreased parasympathetic modulation and reduced cardiac baroreflex sensitivity in both groups
Thuillier et al. (2022) [3]	Prospective, single-center observational study	*N* = 12 Female = 2 Age (years) median 40 (IQR 33–47) BMI (kg/m^2^) median 23.1 (IQR 21.2–25.1)	Type 1 (*N* = 12) HbA_1c_ (%) median 6.8 (IQR 5.5–8.9) Duration (years) median 8 (IQR 0–35) TDI (IU/kg) median 38.5 (IQR 17–63) with MDI (*N* = 10) and CSII (*N* = 2)	Amateur Previous marathon participation (*N* = 10)	Paris marathon (42.195 km) Finishing time (min) median 243.5 (IQR 210–305) Carbohydrate intake median 33.9 g/h (IQR 13.6–73)	CGM (*N* = 12) TIR^c^ 51.6% TBR 0% TAR 48.4%	Glucose kinetics	Mean glucose levels at the start of the race were 13.7 ± 4.2 mmol/l (247 ± 76 mg/dl) and at the end 7.8 ± 2.3 mmol/l (140 ± 41 mg/dl) No hypoglycemic episodes during the race More time spent in hyperglycemia during the race (48.4%) compared to 2-h preparatory run (18.4%) Significantly less time spent in euglycemia during the race (51.6%) compared to 2-h preparatory run (58.0%)
Tuominen et al. (1997a) [28]	Prospective, observational study	*N* = 7 Female = 1 Age (years) 37 ± 3 BMI (kg/m^2^) 23.9 ± 0.5	Type 1 (*N* = 7) HbA_1c_ (%) 7.7 ± 0.3 Duration (years) 16 ± 5 TDI (IU) 20 ± 3 of short acting and 17 ± 2 of intermediate acting insulin MDI (*N* = 7)	Amateur VO_2_max (ml/kg min) 46 ± 1 (range 35–51) Training volume (km/week) 60	Marathon (42.195 km) Carbohydrate intake (g) total 336 ± 18	Manual (*N* = 7)	Metabolic and hormonal changes, insulin sensitivity, muscle glycogen content and glycogen synthase activity	No increase in insulin sensitivity after the race Significant increase in lipid oxidation in the basal state after the race No significant increase in muscle glycogen content the day after the race Moderate reduction in insulin dose prior to the marathon Serum CK levels rose by 5.5-fold immediately after the race up to 25.4-fold 24-h post-race Serum cortisol levels rose by 2.3-fold immediately after the race and GH levels by 1.8-fold with normalization within 24-h post-race
Tuominen et al. (1997b) [17]	Prospective, controlled observational study	*N* = 35 Female = 0 Age (years) T1DM 37 ± 3, marathon control 38 ± 2, treadmill control 27 ± 1 BMI (kg/m^2^) T1DM 23.9 ± 0.5, marathon control 22.8 ± 0.3, treadmill control 22.4 ± 0.4	Type 1 (*N* = 7) HbA_1c_ (%) T1DM 7.7 ± 0.3 Duration (years) range 16 ± 5 TDI (IU) 37 ± 5 U MDI (*N* = 7)	Amateur VO_2_max (ml/kg min) T1DM 46 ± 1, marathon control 48 ± 2, treadmill control 59 ± 2	Marathon (42.129 km), 2-h treadmill run at 75% VO_2_max	Manual (*N* = 7)	Insulin clearance after strenuous exercise	Decreased serum insulin levels post-exercise in both groups Insulin clearance increased in runners with diabetes and healthy controls after exercise Similar urinary insulin excretion in both groups post-exercise Decreased serum triglycerides after exercise with inverse correlation to insulin clearance Significant increase in cortisol immediately after the marathon by 1.3-fold
van Loon et al. (1992) [36]	Prospective, controlled observational study	*N* = 15 Female = 0 Age (years) T1DM 42.7 ± 5.2, control 37.1 ± 9.0	Type 1 (*N* = 7) HbA_1c_ (%) 9.4 ± 1.4 Duration (years) 12.0 ± 5.7 TDI (IU) 37 ± 5 U MDI (*N* = 7)	Amateur Training volume (km/week) T1DM 64 ± 19, controls 58 ± 25 Previous marathon (*N* = 15)	3-h 32 km run	Manual (*N* = 7)	Response of fibrinolytic system to prolonged exercise	Decrease in PAI-1 antigen in both groups Increase of t-PA antigen, vWF and VIII:C in both groups Correlation between t-PA increase and exercise intensity Lower increase of t-PA but larger increase in u-PA in runners with diabetes compared to healthy controls

*BMI* body mass index, *CGM* continuous glucose monitoring, *CK* creatine kinase, *CSII* continuous subcutaneous insulin infusion, *fT3* free triiodothyronine, *fT4* free thyroxine, *GH* growth hormone, *HbA*_*1c*_ glycosylated hemoglobin, *IQR* interquartile range, *MDI* multiple daily injections, *PAI-1* plasminogen activator inhibitor-1, *TAR* time above range, *TBR* time below range, *TDI* total daily insulin dose, *TIR* time in range, *t-PA* tissue-type plasminogen activator, *TSH* thyroid-stimulating hormone, *T1DM* Type 1 diabetes mellitus, *u-PA* urokinase-type plasminogen activator, *VIII:C* factor VIII procoagulant activity, *VO*_*2*_*max* maximal oxygen consumption, *vWF* von Willebrand factor

^a^Hyperglycemia defines as glucose > 10.0 mmol/l (180 mg/dl) [25]

^b^Hypoglycemia defined as glucose < 3.9 mmol/l (70 mg/dl) [25]

^c^Time in range defined as glucose 3.9–10.0 mmol/l (70–180 mg/dl) [25]

**Table 4:** Summary of study and population characteristics, methods and key findings of included case reports and series

Study	Design	Sample characteristics	Diabetes characteristics	Running history	Race characteristics and performance	Glucose monitoring during the race	Key parameters assessed	Main results
Bach et al. (2017) [37]	Case study	*N* = 1 Female = 0 Age (years) 35 BMI (kg/m^2^) 23.8	Type 1 MDI	Amateur Previous marathon participation	3-day Ultra-triathlon (1st day: 10 km swimming, 144.8 km cycling 136 m elevation change, 2nd day: 275.4 km cycling 1340 m elevation change, 3rd day: 84.4 km running 545 m elevation change) Finishing time (h) 29.4 (6th overall)	CGM TIR^a^ Stage 1 (27.4%), Night 1 (24.2%), Stage 2 (17.9%), Night 2 (26.3%), Stage 3 (51.3%) TBR 0% TAR "High (130-240mg/dl): Stage 1 (66.1%), Night 1 (69.7%), Stage 2 (3.4%), Night 2 (73.7%), Stage 3 (15.1%) Hyperglycemic (> 240mg/dl): Stage 1 (6.5%), Night 1 (6.2%), Stage 2 (0%), Night 2 (0%), Stage 3 (0%)"	Glycemic dynamics and metabolic changes	No events of post-exercise or nocturnal hypoglycemia No hypoglycemic events during the competition Good overall glycemic control with TAR 2.2% during the effective exercise Post-exercise and nocturnal blood glucose values were higher or hyperglycemic Sequential increase in CRP and aldosterone Peak CK (by 34.1-fold) and serum cortisol (2.0-fold) values after the race
Belli et al. (2017) [34]	Case report	*N* = 3 Female = 0 Age (years) 35.3 ± 1.5 BMI (kg/m^2^) 23.8 ± 1.0 Body fat (10%) 11.7 ± 2.0	Type 1 (*N* = 3) HbA_1c_ (%) range 6.9–8.7 Duration (years) 23.3 ± 8.0 TDI (IU) 27.6 ± 9.0 CSII (*N* = 1) MDI (*N* = 2)	Elite Training volume (km/week) range 40–100 km Previous marathon participation (*N* = 3)	Relay ultra-marathon (217 km in total, 12,000 positive and negative elevation), completed distance by athletes were 68.7 km, 84.5 km, 65.1 km Finishing time (h) 29.5 (3rd overall)	CGM (*N* = 1) Manual (*N* = 2)	Glycemic control, changes in biomarkers of muscle damage, inflammation and renal function, muscle soreness	Good glycemic control was maintained during the race Increase in serum creatinine, without meeting the criteria for AKI 73% of blood glucose readings were between 90 and 250 mg/dl (5.0–13.9%) where it is safe to exercise Only three asymptomatic hypoglycemic events (> 3.9 mmol/l [< 70 mg/dl]) CK levels were elevated by 9.4-, 9.9-, and 50.8-fold in the three runners immediately after the race
Braune et al. (2019) [32]	Case report	*N* = 1 Female = 0 Age (years) 49	Type 1 Duration (years) 32 CSII with open-source algorithm	Recreational Training volume (km/week) 15 No previous marathon participation	Half-marathon (21.1 km) Finishing time (h) 1.9 Carbohydrate intake (g/h) 12.6	CGM TIR^a^ (%) 100 TBR (%) 0 TAR (%) 0	Glycemic dynamics using an artificial pancreas system	TIR during the race 100% Stable glucose levels post-race with an average glucose level of 6.6 mmol/l (119 mg/dL) and 95.8% TIR on the race day and the following day No late-onset hypoglycemia
Knechtle and Nikolaidis (2017) [9]	Case report	*N* = 1 Female = 0 Age (years) 64 BMI (kg/m^2^) 21	Type 1 (LADA) Duration (years) 1 TDI (IU) 35–45 MDI	Amateur Training volume (km/week) 82 Previous marathon participation	Marathon	CGM	Glucose dynamics	Positive correlation between decrease in blood glucose and running duration as well as running pace Reduction of basal insulin rate by 20–50% at race day
Knechtle and Nikolaidis (2018) [15]	Case report	*N* = 1 Female = 0 Age (years) 63	Type 1 HbA_1c_ (%) 6.0 Duration (years) 42 TDI (IU) 35–45 CSII	Elite Training volume (km/week) 170 Previous marathon participation	24-h ultra-marathon Performance (km) 133	CGM	HbA_1c_ in relation to training volume	Significant increase in monthly training mileage Significant decrease of HbA_1c_ during the years Significant association between monthly training mileage and HbA_1c_
Mondtt-Blanchard et al. (2025) [31]	Case report	*N* = 1 Female = 1 42–47 years (at time of each marathon	Type 1 HbA_1c_ (%) 6.9 ± 0.2 Duration (years) 37–44 CSII	Recreational Previous marathon participation	Marathon (42.195 km) Finishing time (min) 249 ± 15.4 Carbohydrate intake (g/kg/h) 0.2–0.4	CGM TIR (%): 59–75 (Race 1); NA (Race 2); 57–87 (Race 3); 35–96 (Race 4) TBR (%) 3–6 (Race 1); NA; 0–5 (Race 3); 0–1 (Race 4 TAR (%): 33–17 (Race 1); NA; 23–4 (Race 3); 39–1 (Race 4)	Technological challenges of CGM in marathon running	CGM signal interference occurred in all 4 marathons (15–100% of race time), compromising AID system function and glycemic management Adaptability and pre-established contingency plans are essential for safe race completion
Onetto et al. (2025) [30]	Case report	*N* = 3 Female = 1 Age (years) 40.3 ± 8.7	Type 1 (*N* = 3) HbA_1c_ (%) 6.7 ± 0.2 TDI (IU) 33.8 ± 5.0 CSII (*N* = 3)	Amateur Previous marathon participation (*N* = 2)	Marathon (42.195 km) Finishing time (min) 186.7 ± 42.2 Carbohydrate intake (g/kg/h) C1: 0.39 (101 g total); C2: 0.42 (120 g total); C3: 0.50 (115 g total)	CGM (*N* = 3) TIR^a^ (%):C1: 96%; C2: 100%; C3: 0% TBR (%) C1: 4; C2: 0; C3: 0 TAR (%) C1: 0; C2: 0; C3: 100	Glycemic control strategies	AID systems can support safe marathon completion Individualized education from a multidisciplinary team is essential; carbohydrate intake of 0.3–0.5 g/kg/h should be personalized and tested during training runs
Silva Daniel et al. (2022) [33]	Case report	*N* = 1 Female = 0 Age (years) 36 BMI (kg/m^2^) 25.8 Body fat (%) 10	Type 1 HbA_1c_ (%) 5.7 Duration (years) 45 TDI (IU) 35–45 MDI	Elite Training volume (km/week) 40–100 Previous marathon participation	217-km ultra-marathon Finishing time (h) 51.3 Carbohydrate intake (g/h) 10.4	Manual TIR^a^ (%) 47 TBR (%) 5 TAR (%) 16 (> 10–13.9 mmol/l [180–250 mg/dl]), 32 (> 13.9 mmol/l [250 mg/dl])	Nutritional strategies and glycemic response	Total consumption of 15.0 MJ (3593 kcal), 532 g of carbohydrates, 166 g of protein, 92 g of fat, and 14 L of water during the 217-km ultra-marathon Glycemic values varied significantly, with most values being in TIR 5% of total blood glucose readings were hypoglycemic
van den Boom et al. (2021) [7]	Case report	*N* = 1 Female = 1 Age (years) 43	Type 1 HbA_1c_ (%) 6.2 Duration (years) 16 CSII (hybrid-closed-loop system)	Amateur Training volume (km/week) 40 No previous marathon participation	Half-marathon (21.1 km) Finishing time (h) 2.3 Carbohydrate intake (g/h) 39.1	CGM TIR^a^ (%) 55.6 TBR (%) 0 TAR (%) 44.4	Glycemic control using a hybrid closed-loop system	Successful completion of the half-marathon using hybrid-closed loop system without any hypoglycemic events No late-onset hypoglycemia 24-h post-run Hypoglycemia (< 3.0 mmol/l [54 mg/dl]) 0.2% within 48-h post-run
Vlahek et al. (2013) [38]	Case report	*N* = 1 Female = 0 Age (years) 27	Type 1 HbA_1c_ (%) 5.5 Duration (years) 21 TDI (IU) 25–30 MDI	Recreational Previous marathon participation	Double Ironman (7.6 km swimming, 360 km cycling, 84.4 km running) Finishing time (h) 29.2	Manual	Glycemic dynamics and biomarker changes	Safe completion of a double Ironman by a T1DM patient Increase in biomarkers of inflammation, kidney damage, muscle damage, and liver damage in line with values from the literature
Weiss et al. (2023) [10]	Case report	*N* = 1 Female = 0 Age (years) 66	Type 1 (*N* = 1) Duration (years) 15 TDI (IU) 35–45 CSII (*N* = 1)	Elite Training volume (km/week) 138 Previous marathon participation (*N* = 1)	6-day ultra-marathon Total distance covered (km) 467.4	CGM (*N* = 1)	Evolution of training volume and HbA_1c_ levels	Completion of 48 24-h ultra-marathons with Type 1 diabetes mellitus No significant hypoglycemia during competition over the years HbA_1c_ levels correlated significantly with monthly running kilometers

*AKI* acute kidney injury, *BMI* body mass index, *CGM* continuous glucose monitoring, *CK* creatine kinase, *CRP* C-reactive protein, *CSII* continuous subcutaneous insulin infusion, *HbA*_*1c*_ glycosylated hemoglobin, *LADA* latent autoimmune diabetes in adults, *MDI* multiple daily injections, *TAR* time above range, *TBR* time below range, *TDI* total daily insulin dose, *TIR* time in range

^a^Time in range defined as glucose 3.9–10.0 mmol/l (70–180 mg/dl) [25]

### Diabetes Characteristics and Glucose Monitoring

In total, 99 participants (61.5%) had diabetes, and 62 (38.5%) patients were healthy controls. Regarding participants with diabetes, Type 1 diabetes mellitus was present in 98 participants (99.0%, including one late-onset autoimmune diabetes in the adult), and Type 2 diabetes mellitus in 1 participant [1.0%]). Regarding glucose monitoring, 13 studies (59.1%) examined CGM [3, 7–10, 15, 20, 27, 29–32, 34, 37]. In total, 50 participants with diabetes (50.5%) used CGM for glucose measurements, 33 (33.3%) measured manually (especially in studies published before 2010), and measurement technique was not specified in 16 (16.2%). On the other hand, 27 participants with diabetes (27.2%) used continuous subcutaneous insulin infusion (CSII) for insulin application, 63 (63.6%) practiced multiple daily injection (MDI), and method of insulin application was not specified in 7 (7.0%). The reported range for HbA_1c_ in studies that gave values was 5.5–10.6%, with an overall weighted mean of 7.4% (95% CI 6.9–8.1) (*N* = 15) [3, 7, 8, 15, 17, 20, 27–31, 33–36, 38]. The weighted mean diabetes duration was 14.5 (95% CI 12.4–16.7) years (*N* = 15) with a range of 0.3–45 years [3, 7–10, 15–17, 20, 28, 29, 32–36, 38].

### Glycemic Control and Monitoring of Late-Onset Hypoglycemia

Several studies reported blood glucose dynamics across different race distances as summarized in Tables 3 and 4. To enhance clarity, we categorize findings based on TIR, TBR, and TAR.

In half-marathon events, TIR among individuals with diabetes using CGM ranged from 40 to 100% [7, 20, 32]. For marathon, one study reported TIR of 51.6% [3], while another observed two asymptomatic hypoglycemic events [27]. Another study comparing glycemic variability between a marathon and 2-h preparatory runs found that TIR was significantly lower during the marathon [3]. In longer endurance events, such as ultra-marathons and triathlons, TIR values varied widely. In ultra-marathons, TIR was reported at 47–73% [33, 34], whereas in ultra-triathlons, it was reported as between 17.9 and 51.3% [37].

Data on hypoglycemia during half-marathons are limited, as no studies reported TBR values, and one study did not measure blood glucose levels at all during the race [11]. In marathons, one study found that no time was spent in hypoglycemia, either during the race or in preparatory runs [3]. Three of five marathon runners with diabetes experienced symptomatic hypoglycemia, including one episode undetected by CGM [29]. In runners using hybrid closed-loop systems, TBR ranged from 0 to 4%, with one runner developing hypoglycemia symptoms at kilometer 40 [30]. However, another study reported two asymptomatic hypoglycemic events [27]. In ultra-endurance events, hypoglycemia was more frequently observed. In ultra-marathons, TBR was documented at 5%, with two and three asymptomatic hypoglycemic events recorded in different studies [33, 34]. In ultra-triathlons, low blood sugar (< 4.4 mmol/l [80 mg/dL]) was reported for 0–78.6% of the race time [37].

In half-marathons, TAR varied considerably, ranging from 0 to 44% of the race duration [7, 20, 32]. During marathons, a study comparing marathon and preparatory runs found that participants spent significantly more time in hyperglycemia (> 11.1 mmol/l [> 200 mg/dL]) during the marathon than in the final preparatory run [3]. Another study observed one hyperglycemic event [27], with one runner using a hybrid closed-loop system spending 53% of the marathon above 13.9 mmol/l (250 mg/dl), attributed to insufficient pre-race insulin and misuse of the temporary target mode [30]. Also CGM signal interference during 15–100% of race time repeatedly compromised automated insulin delivery, contributing to hyperglycemia up to 16.7 mmol/l (301 mg/dl) [31]. In ultra-endurance events, hyperglycemia was also common. TAR reached 48% in ultra-marathons [33], with five hyperglycemic events noted [8]. In the IRONMAN^®^ triathlon, five hyperglycemic incidents were observed, primarily in the first half of the cycling phase [16]. Similarly, in ultra-triathlons, high blood sugar (7.2–13.3 mmol/l [130–240 mg/dL]) was reported for 3.4–69.7% of the race time, while severe hyperglycemia (> 13.3 mmol/l) was recorded for 0–6.5% of the race duration [37].

Two studies examining glycemic variability, measured as coefficient of variation (CV) in blood glucose levels using CGM, found significant fluctuations during the race of a half-marathon and a marathon [20, 27]. Notably, glycemic variability was significantly lower during the competition compared to pre- and post-race states [20]. Athletes with diabetes, however, showed significantly higher glycemic variability and significantly lower TIR compared to healthy controls [20]. Comparing glucose pattern between individuals with diabetes and healthy controls pre-, during and post-competition, a similar sensor glucose pattern was found between the groups, with athletes with diabetes having higher blood glucose levels overall [20].

Regarding the monitoring of late-onset hypoglycemia defined as hypoglycemic episodes generally occurring 6–15 h after strenuous exercise in individuals with diabetes [39], five studies (26.3%) [7, 20, 27, 32, 37] performed observational measurements using CGM of athletes during the night up to 48-h after the race. A total of 27 runners with diabetes were observed for late-onset glycemic imbalances. Only one study reported a TBR of 0.2% (< 3 mmol/l [54 mg/dl] 48-h post-race in one athlete using CGM measurement [7]. Other studies providing TIR 24-h after the race showed results ranging between 24 and 94.2% [7, 20, 37], TBR (3.0–3.8 mmol/l [54–68 mg/dl]) 0.7–2% [7, 20], TAR 1 (> 10.0–13.9 mmol/l [180–250 mg/dl]) 5.1–74% [7, 20, 37], and TAR 2 (> 13.9 mmol/l [> 250 mg/dl]) 0–6.2% [7, 20, 37]. One study observed a hyperglycemic episode in the morning after the race exceeding a blood glucose level of 19.4 mmol/l (350 mg/dl) [27], whereas one study did not record hypoglycemic or hyperglycemic events [32].

### Insulin Management

Overall, 18 (81.8%) studies reported on insulin adjustments before and during the race. The most commonly used insulin adjustment was a 50% reduction of basal insulin either 24-h before or on race-day reported by five studies (22.7%) [3, 7, 16, 29, 32]. Four studies (18.2%) observed a reduction of basal insulin application by 70–80% before the race [8, 10, 15, 31]. Continuation of the same application scheme as usual [33, 37, 38], complete omission of basal insulin on race day [9, 35, 36] and basal insulin reduction between 5 and 35% before the race [28, 30, 34] were described by three studies (13.6%).

### Nutritional Intake and Changes in Metabolism

Nutrition undoubtedly plays an important role in endurance sports performance. Ten studies (45.5%) reported on carbohydrate intake during competitions. During half-marathon races, carbohydrate intake ranged from 12.6 to 39.1 g/h for individuals [7, 20, 32] while healthy controls consumed 11.5 g/h in one study [20]. Similarly, athletes running a marathon consumed carbohydrates at a rate between 25 and 33.9 g/h [3, 28, 30, 31]. Carbohydrate consumption during a 82-km mountain ultra-marathon was 33.9 g/h [8], while during the 217-km ultra-marathon 10.4 g/h was consumed [33] and during an Ironman^®^ a range between 24 and 60 g/h was observed [16].

Various biomarkers exhibited significant changes following exercise (see Tables 3 and 4). Creatine kinase (CK), a marker of skeletal muscle damage, has been reported to increase between 5.5- and 50.8-fold immediately post-exercise [28, 34, 37, 38], peaking at 24 h post-race [28] and returning to baseline within seven days [38]. Additionally, stress hormone levels also rise transiently. Serum cortisol concentrations increase by 1.3- to 2.3-fold immediately after the race [17, 28, 37], normalizing within 24 h [28]. Similarly, growth hormone (GH) levels elevate by approximately 1.8-fold post-race before returning to baseline within the same period [28]. These biomarker fluctuations reflect the physiological stress and recovery process associated with endurance exercise.

## Discussion

**Fig. 2:**
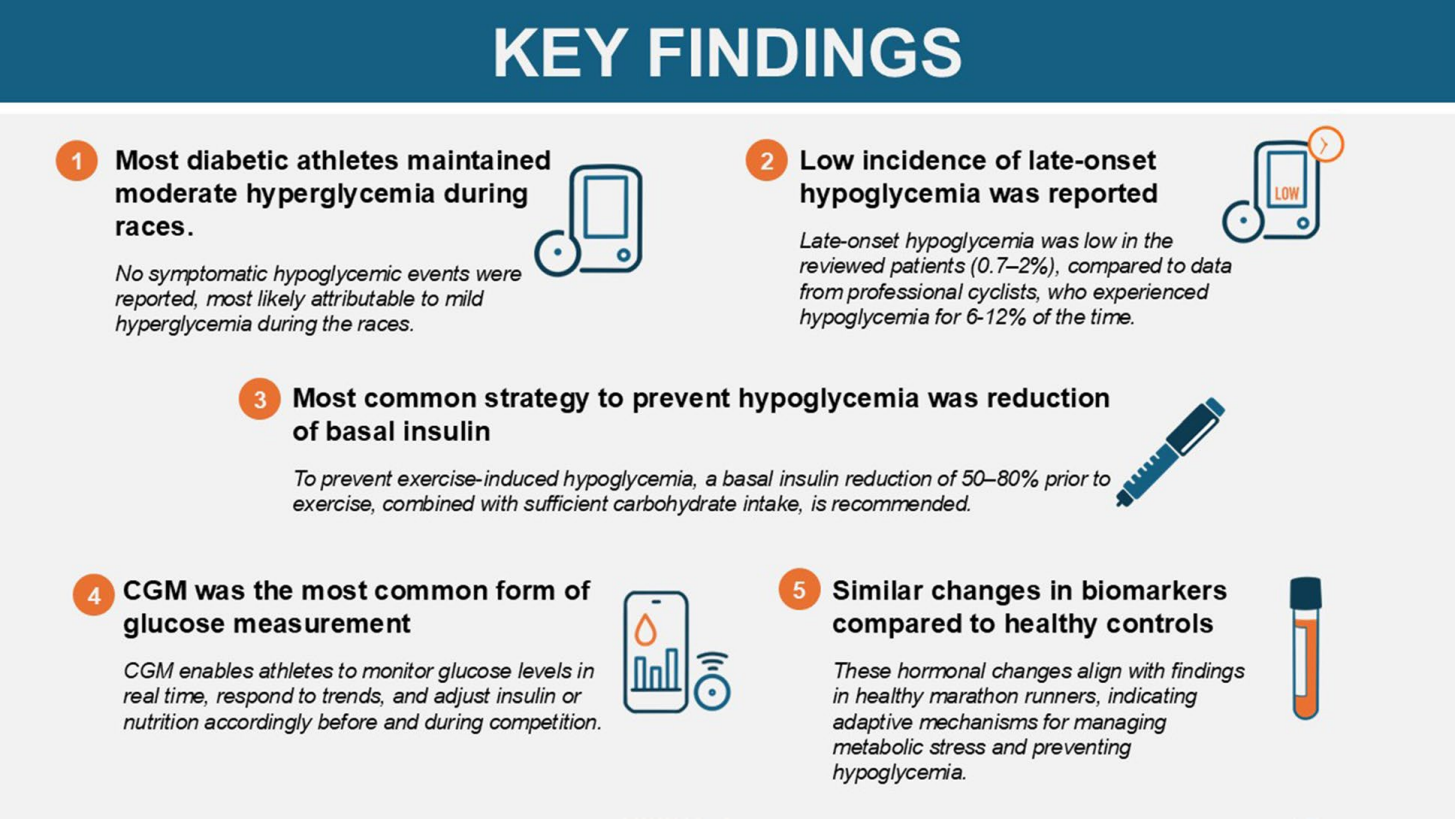
Key findings of the systematic review. *CGM* continuous glucose monitoring

In this systematic review, we comprehensively assessed the current literature about long-distance athletes with diabetes. The key findings of this systematic review on diabetes mellitus and long-distance running showed that (1) long-distance runners with diabetes maintained moderate hyperglycemia to prevent hypoglycemia during races; (2) a relatively low incidence of late-onset hypoglycemia was reported; (3) the most common strategy to prevent exercise-induced hypoglycemia was a basal insulin reduction of 50–80%; (4) CGM use was the most common form of glucose monitoring and (5) biomarkers of stress-related hormonal changes responded similarly compared to healthy controls (Fig. 2).

### Differences in Glycemic Control

The vast majority of the study population with diabetes included in this systematic review consisted of runners with Type 1 diabetes mellitus, while only one participant had Type 2 diabetes mellitus. Overall, regular glycemic control in the study population with diabetes was fairly well, although the weighted mean HbA_1c_ was 7.4% (95% CI 6.9–8.1), slightly above the recommended goal of less than 7% [40]. This observation may align with existing evidence suggesting that regular exercise improves HbA_1c_ levels [41, 42], though a direct causal inference cannot be drawn from the data of this review alone. Given that participants in competitive long-distance running events are typically well-trained, their observed HbA_1c_ of 7.4% is consistent with the beneficial effects of aerobic exercise on glycemic control in Type 1 diabetes mellitus [41, 42]. Notably, this HbA_1c_ level is lower than the average observed in the general diabetic population, further supporting the positive impact of regular endurance training [43]. Conversely, effective long-term glucose management enhances performance in athletes with Type 1 diabetes, as lower HbA_1c_ levels (~ 6.5%) are associated with better cardiorespiratory fitness and pulmonary function compared to higher HbA_1c_ levels (~ 7.8%) [44]. Many of the elevated HbA_1c_ values observed are linked to older studies included in this review. When focusing solely on studies published after 2010, the weighted mean HbA_1c_ was 6.7% (95% CI 6.9–8.1) compared to 8.3% (95% CI 7.4–9.3) in studies published before 2010 with statistical significance (*p* = 0.002 in the Q-test for heterogeneity between groups), indicating exceptional glycemic control. This improvement may be attributed to advancements in diabetes management technologies, such as CGM, insulin pumps, and automated insulin delivery (AID) systems. Moser et al. observed that athletes with diabetes using CGM maintained stable glucose concentrations throughout the running competition, with an absence of significant hypoglycemic or hyperglycemic excursions, demonstrating effective glycemic management [20]. One possible hypothesis is that some athletes and their clinicians may accept a less stringent HbA_1c_ target to reduce the risk of hypoglycemic events during training, races, and daily life, though this warrants further investigation.

It is conceivable that glycemic control varies with regard to race distance (e.g., half-marathon vs. marathon), race intensity (e.g., finishing time) and race modality (e.g., marathon vs. triathlon). Most studies reported similar TIR between the different race distances and modalities. Interestingly, most athletes with diabetes spent a significant amount of time in hyperglycemia, while no symptomatic hypoglycemic events were reported and generally only a very small number of hypoglycemic events were observed. Most likely, athletes with diabetes intentionally aim to run in moderate hyperglycemia to prevent hypoglycemic events during competition.

Naturally, stress hormones such as cortisol and epinephrine will be released during long-distance running such as a marathon. Studies showed increased levels of adrenocorticotropic hormone, cortisol, growth hormone, catecholamines and glucagon levels during the competition [16, 35]. The release of these hormones inevitably contributes to a rather hyperglycemic metabolism during the run. Interestingly, one study showed significantly higher plasma glucagon levels in subjects with diabetes compared to control subjects, despite similar blood glucose levels between the two groups [35]. This finding may reflect a compensatory mechanism; in individuals without diabetes, the intact glucose–insulin feedback loop is sufficient to prevent hypoglycemia during exercise, whereas in athletes with Type 1 diabetes mellitus an exaggerated glucagon response may represent the primary available counterregulatory defence against exercise-induced hypoglycemia. In contrast, studies on healthy marathon runners have reported elevated blood glucose levels, along with significantly increased cortisol and catecholamine levels immediately after completing a marathon. Blood glucose levels normalized within 24 h; cortisol levels, measured in morning samples, similarly returned to baseline within one day, though diurnal variation should be considered when interpreting these findings [45].

### Late-Onset Hypoglycemia

Late-onset or nocturnal hypoglycemia after exercise in individuals with diabetes is a well-documented phenomenon, particularly in Type 1 diabetes [46, 47]. Recent studies using CGM in professional athletes with Type 1 diabetes mellitus, primarily cyclists with diabetes, indicate that while in-race glycemia is well managed, the risk of hypoglycemia significantly increases during the post-exercise nighttime period, particularly after evening exercise sessions or during training camps [48]. This delayed hypoglycemia, which can occur 6–28 h after vigorous exercise, is largely attributed to higher insulin sensitivity, increased glucose uptake and glycogen synthesis in previously exercised muscles, driven by elevated glucose transport via glucose transporter type 4 (GLUT-4) [20, 27, 49, 50]. The incidence of late-onset hypoglycemia in the patients included in this review seems to be relatively low, ranging from 0.7 to 2%, in contrast to data from professional cyclists, where the time spent in hypoglycemia ranges from 6 to 12% [51]. A possible explanation for this difference might be due to differences in exercise intensity, duration, insulin adjustments and nutritional intake, such as carbohydrate supplementation during and after exercise. However, data on runners remain scarce, highlighting a need for further research into how these dynamics may differ across athletic disciplines.

Interestingly, this phenomenon has also been observed in individuals without diabetes, though the underlying mechanisms remain unclear [20, 52, 53]. On the other hand, some studies in ultra-endurance exercise have reported hyperglycemia during post-exercise periods, potentially linked to prolonged exercise-induced muscle damage or stress-related hormonal changes like cortisol and catecholamine release [37].

The risk of nocturnal hypoglycemia can vary based on different factors such as exercise timing, post-exercise carbohydrate intake, and insulin administration [46]. The risk of late-onset hypoglycemia generally remains elevated for 15–24 h post-exercise, with a significantly lower incidence observed following morning exercise sessions compared to those in the afternoon [54]. Additionally, inadequate carbohydrate intake following prolonged exercise may delay muscle and liver glycogen restoration, potentially increasing the risk of nocturnal hypoglycemia [55, 56]. For athletes with Type 1 diabetes, promptly and sufficiently replenishing muscle and liver glycogen stores is crucial to mitigate the risk of late-onset hypoglycemia [46]. Beverages containing both carbohydrates and protein can support post-exercise recovery and help prevent delayed hypoglycemia [57]. Additionally, a single caffeine dose (5–6 mg/kg body mass) has been shown to reduce exercise-induced glycemic decline but is associated with elevated bedtime glucose levels and lower early morning glucose concentrations compared to placebo [58]. Strategies to mitigate this risk include using CGM for continuous monitoring and adjusting carbohydrate intake or insulin therapy based on post-exercise needs. CSII appears superior to MDI in managing exercise-related glycemic fluctuations, offering greater flexibility in basal insulin adjustments to reduce both early and late-onset hypoglycemia [46]. Additionally, CSII has been shown to limit post-exercise hyperglycemia without increasing the risk of late-onset hypoglycemia [59, 60]. Possible strategies to reduce late post-exercise hypoglycemia include implementing a 20% reduction in overnight basal insulin, which has been shown to effectively raise nocturnal blood glucose levels in children and adults with Type 1 diabetes and decrease hypoglycemia risk [59, 61]. Elevated insulin sensitivity after exercise can be addressed through a reduction of about 50% in the bolus insulin dose administered at the meal after exercise, along with consumption of a snack with a low glycemic index at bedtime [62]. Individuals at elevated risk for severe nocturnal hypoglycemia, such as those with frequent episodes of hypoglycemia or who sleep alone, should implement additional precautions, such as checking blood glucose levels during early morning hours or using a real-time CGM with alarms and automatic pump suspension or AID systems [46, 63]. Merely consuming a snack without adjusting basal insulin does not completely eliminate the risk of nocturnal hypoglycemia, and alcohol intake may further increase this risk.

Additionally, further research is warranted to explore the effects of exercise duration, intensity, and insulin dosing on post-exercise glycemic control, particularly for recreationally active individuals with Type 1 diabetes who may have less experience managing exercise-related therapy adjustments.

### Prevention Strategies for Exercise-Induced Hypoglycemia

During endurance exercise, insulin secretion decreases in individuals without diabetes due to sympathetic activation, which releases catecholamines that inhibit pancreatic beta cells via alpha-adrenergic receptors [63, 64]. Simultaneously, muscular activity enhances glucose uptake through GLUT-4 translocation, reducing the need for insulin-mediated glucose uptake [65]. These adaptations ensure energy availability while minimizing hypoglycemia risk [63–65]. During early recovery after intense exercise, insulin secretion rises to balance decreased glucose utilization and consecutively increasing glucose levels. In individuals with Type 1 diabetes, insulin levels depend mainly on the administration of exogenous insulin, and they often experience hyperinsulinemia during exercise, increasing the risk of hypoglycemia by suppressing fat oxidation and enhancing glucose utilization. Intensive exercise can also exacerbate post-exercise hyperglycemia due to the inability to adjust insulin delivery automatically, and omitting insulin prior to exercise can lead to excessive hyperglycemia and ketone production [46, 63, 66].

The most important goal for athletes with diabetes is the prevention of exercise-induced hypoglycemia. In the studies in this review, no symptomatic hypoglycemic events were reported. Both intrinsic and extrinsic factors take part in glycemic control of subjects with diabetes. On the one hand, several metabolic counterbalance mechanisms are in place to prevent diabetic runners from developing hypoglycemia. As such, hepatic gluconeogenesis significantly increased and levels of growth hormone, glucagon, cortisol, adrenocorticotropic hormone (ACTH), and catecholamines were significantly elevated during the race [16, 35].

Glycemic targets for athletes with Type 1 diabetes mellitus should be personalized in collaboration with healthcare professionals, including specialized endocrinologists and sports physicians, through a shared decision-making approach [63]. According to guidelines, it is recommended to aim for > 70% TIR between 3.9 and 10.0 mmol/l, with less than 4% below 3.9 mmol/l and < 1% below 3.0 mmol/l, in adult individuals with Type 1 diabetes [67]. During competition, athletes should strive for < 1% time below target and > 75% TIR to minimize the impact of hypoglycemia on performance [63, 68]. Additionally, maintaining a coefficient of variation ≤ 36% for CGM values is advised to reduce the risk of hypoglycemia [63, 69]. While these targets are challenging, they may be attainable through advanced technologies and sustained effort [63].

The current literature proposes different insulin adjustment strategies for competitive athletes. For athletes using CSII, a basal rate reduction of 50–80% is recommended starting 1.5 h before exercise [70], while those on MDI are advised to reduce basal insulin by 20–50% [63]. In addition to or as an alternative to insulin reduction, consuming carbohydrates (up to 70–90 g/h) during prolonged aerobic activities can help prevent hypoglycemia and maintain performance [63, 71]. Supporting these strategies, Moser et al. observed that basal insulin reduction effectively mitigated glycemic instability during a running event, emphasizing the importance of insulin adjustments both before and during exercise to prevent hypoglycemic episodes [20].

Carbohydrate intake ranging from 0.4 to 1.3 g per kg body mass per hour has been documented for athletes with Type 1 diabetes engaging in performance activities lasting 60 min or longer [63, 72], a range that closely aligns with the recommendations for healthy marathon runners, who are advised to consume approximately 90 g/h for optimal performance [45]. Studies indicate that this intake range effectively prevents hypoglycemia and enhances endurance performance during prolonged exercise [63]. Interestingly, nearly all participants with diabetes failed to meet the recommended carbohydrate intake during the race, probably to avoid the need for additional insulin administration or hyperglycemia. However, no symptomatic hypoglycemic events were reported (see Tables 3 and 4).

### CGM in Long-Distance Runners with Diabetes

The majority of participants with diabetes including in this systematic review used CGM as a tool for glucose monitoring. CGM allows athletes to track glucose levels in real-time, respond to trend alerts, and optimize insulin therapy [27, 63, 73]. Athletes have the capacity to rapidly respond to fluctuations in blood glucose levels, such as drops or rises, and consequently adjust their nutritional intake in the period leading up to and including competition. For instance, the extant literature suggests the utilization of sugar-free hydration in instances of elevated blood glucose levels (greater than 10.0 mmol/l [180 mg/dl]), while recommending beverages containing carbohydrates in cases where blood glucose levels decline below 8.0 mmol/l (144 mg/dl) [63]. Especially when combined with insulin pumps, CSII offers significant advantages over MDI for athletes with Type 1 diabetes, providing greater flexibility in adjusting basal insulin rates to manage exercise-related changes in glucose levels [74] and provides benefits in managing both early and late-onset hypoglycemia following exercise [46]. CSII allows for reductions in basal insulin before or after prolonged aerobic exercise, increases for intensive work, and adjustments overnight to prevent nocturnal hypoglycemia [46, 59, 60]. AID systems (i.e., hybrid closed-loop systems), which adjust insulin delivery based on current glucose levels and predictions, improve TIR [75] and support better glycemic management during prolonged exercise [74], although challenges such as sensor accuracy and device comfort during exercise such as catheter displacement remain [76]. On the other hand, hybrid closed-loop systems offer practical advantages, as athletes have to carry less blood glucose monitoring equipment and avoid removing gloves for measurements in colder weather [10].

Overall, several articles included in this systematic reviews provided evidence that hybrid closed-loop systems are feasible and safe options for athletes with Type 1 diabetes to complete and participate in several endurance events ranging from half-marathon to ultra-marathons [3, 7, 8, 32].

In recent years, there has been a marked increase in scientific interest in the use of CGM for runners without diabetes. CGM has been shown to be useful in capturing glucose trends during prolonged endurance activities [76]. Athletes without diabetes have experienced post-exercise hyperglycemia with a gradual return to normal levels, demonstrating dynamic metabolic changes [76]. CGM has been demonstrated to capture rapid glucose fluctuations linked to exercise intensity, offering insights that extend beyond those obtained through traditional monitoring [76]. During high-intensity training, CGM detected both hypoglycemic and hyperglycemic episodes in athletes without diabetes [77]. Maintaining glycemic stability during prolonged high-intensity exercise without carbohydrate intake was challenging [77]. Athletes who used CGM-informed strategies improved glucose management compared with those relying solely on subjective cues [77]. Furthermore, CGM effectively monitored glucose metabolism during a 100-km ultra-marathon, highlighting adaptive glucose uptake in athletes without diabetes [78]. Continuous monitoring facilitated the optimization of carbohydrate replenishment strategies, thereby preventing energy depletion during prolonged events [78]. Additionally, athletes utilizing CGM were able to anticipate energy lows and make timely adjustments, which may have led to enhanced performance [78].

CGM offers valuable insights into glucose fluctuations and tracking immediate metabolic responses for non- athletes with diabetes, helping coaches and athletes fine-tune carbohydrate intake and optimize both in-session fueling and post-exercise recovery [76–78]. By addressing glycemic variability, CGM can potentially prevent energy crashes and maintain steady athletic performance [76–78]. However, further research is necessary to validate its accuracy under different exercise conditions and to develop algorithms tailored specifically for athletic performance.

### Metabolic and Biomarker Changes

Long-distance endurance exercise induces significant metabolic changes in athletes with diabetes, affecting insulin sensitivity, clearance, and substrate utilization as the body adapts to prolonged physical stress [17, 28]. Tuominen et al. found no improvement in whole-body insulin sensitivity in patients with diabetes post-race (see Table 3) despite glycogen depletion and increased glycogen synthase activity, suggesting that elevated lipid oxidation may inhibit glucose uptake [28]. This contrasts with traditional expectations that glycogen-depleting exercise enhances insulin sensitivity [79, 80]. Acute exercise notably improved insulin sensitivity immediately after activity, while regular training led to sustained benefits [81]. Endurance training enhanced insulin sensitivity through increased lipid oxidation and improved mitochondrial adaptation [82], while resistance training boosted glucose uptake and insulin sensitivity independently of weight loss [44]. Elevated post-exercise insulin clearance may help prevent post-exercise hypoglycemia by reducing circulating insulin levels [81]. Separately, long-term insulin demand is lowered due to improved insulin sensitivity, which reduces the amount of insulin required for glucose regulation [81]. Enhanced glucose uptake by skeletal muscles was linked to increased insulin-independent GLUT-4 translocation [44, 81], improved mitochondrial efficiency [81, 82], and greater capillary density and blood flow [82]. Reduced circulating free fatty acids contributed to improved insulin signaling [82], while increased skeletal muscle mass promoted better glucose disposal and insulin receptor activation [44]. Post-exercise improvements were further supported by increased glycogen storage capacity and a reduction in inflammatory markers, collectively enhancing insulin sensitivity [44].

In a different study, insulin clearance increased significantly in both healthy and subjects with diabetes following strenuous exercise, accompanied by decreased plasma insulin concentrations during insulin infusion [17]. The observed rise in insulin clearance, despite elevated free fatty acid levels, indicates additional mechanisms—possibly the enhanced activity of insulin-degrading enzymes—that regulate insulin metabolism post-exercise [17].

The findings have important implications for endurance athletes, particularly those with diabetes. While increased glycogen synthase activity suggests a potential for glycogen restoration, the combination of elevated lipid oxidation and the need for precise insulin management highlights the importance of tailored post-exercise nutritional strategies [17, 28]. Contrary to the initial assumption of unchanged insulin sensitivity, evidence from randomized controlled trials indicates that regular exercise significantly enhances insulin sensitivity, with effects persisting beyond 72 h after the last exercise session [17, 28, 83]. Furthermore, the increase in insulin clearance suggests a need for tailored insulin dosing during recovery to prevent dysglycemia [17, 28].

Athletes with diabetes are encouraged to incorporate both endurance and resistance training into their routines, as endurance exercise enhances insulin sensitivity and metabolic flexibility, while resistance training improves glucose uptake through muscular and metabolic adaptations [44, 81, 82]. A structured, long-term exercise plan combining both types of activities can significantly improve glycemic control and support overall metabolic health.

The results of the included studies suggest that individuals with Type 1 diabetes exhibit largely similar cardiovascular function to healthy individuals during endurance exercise. However, they face specific challenges in regulating blood pressure and endothelial function (see Table 3) [11, 36]. The blunted response of tissue-type plasminogen activator (t-PA) [36] and the more pronounced post-exercise hypotension observed in individuals with diabetes indicate that their cardiovascular system may struggle more with recovery after intense exercise compared to healthy individuals [11]. However, the increase in t-PA observed in the control group was less pronounced than in other studies investigating healthy marathoners, suggesting that the findings should be interpreted with caution, as factors like training and exercise intensity could influence the results [45]. The impairments in blood pressure regulation, coupled with potential deficiencies in fibrinolysis, emphasize the need for careful monitoring during and after exercise, especially during recovery phases [11]. While the increased urokinase-type plasminogen activator (u-PA) response may help compensate for the t-PA deficiency, there may be a transient risk of decreased fibrinolysis immediately after exercise [11]. However, given the well-documented cardiovascular benefits of exercise in individuals with diabetes [41, 42, 83], the long-term impact of these alterations remains unclear and warrants further investigation.

Numerous studies have documented biomarker alterations following long-distance endurance exercise. Creatine kinase (CK) levels were found to rise immediately after exercise, peaking 24 h post-exercise (see Tables 3 and 4) [28, 34, 37, 38]. Comparisons between subjects with diabetes and healthy marathon runners revealed no significant differences in CK elevation [45]. Similarly, growth hormone (GH) levels increased post-exercise, with most studies reporting comparable changes between and athletes with and without diabetes [16, 17, 28], though one study indicated a greater rise in participants with diabetes [35]. Stress-related hormonal responses, including elevations in catecholamines [35], ACTH [16], and cortisol [16, 17, 28, 37], were observed following strenuous endurance activities. These hormonal changes were consistent with data from healthy marathon runners and suggest similar adaptive mechanisms to manage exercise-induced metabolic stress and hypoglycemia prevention [45].

Collectively, these studies underscore the intricate metabolic alterations triggered by endurance exercise and underscore the necessity for customized management strategies in athletes with diabetes to ensure metabolic stability and optimal performance outcomes.

### Strengths and Limitations

This systematic review has some limitations that constrain the generalizability of its findings. Firstly, the paucity of available data on long-distance runners with diabetes results in relatively small sample sizes across the included studies. Additionally, it is crucial to acknowledge the potential for selection bias within each study, as participants were generally well-controlled in terms of their HbA_1c_. It is plausible that only the most physically fit individuals with diabetes, those with well-managed glycemic control (i.e., stable blood glucose levels and low risk of hypoglycemia) and generally healthy lifestyle, engage in strenuous physical activities such as long-distance running. Moreover, the preponderance of participants with Type 1 diabetes, with only a single case of Type 2 diabetes, is noteworthy, considering the global prevalence of Type 2 diabetes. Consequently, the population with diabetes examined in this review may not be a precise representation of the broader demographic of individuals living with diabetes. Furthermore, only 27.2% of the included studies were rated as high quality, with the majority classified as low or moderate quality, thereby further compromising the robustness of the findings. The lack of consensus in definitions and cut-off values for hyperglycemia and hypoglycemia across studies further complicates the comparison between them. Furthermore, the heterogeneous nature of long-distance runners, encompassing distinct categories such as half-marathoners, marathoners, ultra-marathoners, and ultra-triathletes, complicates the establishment of universal conclusions. While these athletes share certain characteristics, the varying race formats, locations, and requirements introduce significant variability that warrants cautious interpretation of comparisons across different race types. Additionally, all studies reporting on late-onset hypoglycemia exclusively used CGM for post-race glycemic monitoring. Conclusions regarding late-onset glycemic imbalances are therefore limited to CGM users, and may not be generalizable to athletes with diabetes who rely on conventional blood glucose monitoring methods. Finally, technological advancements in diabetes care, such as CGM and CSII systems, introduce additional complexity to the interpretation of these findings. A direct comparison of studies that employ older, manual methods for blood glucose measurements and insulin adjustments with those that incorporate modern technologies may have limited validity due to these advancements in diabetes management. Furthermore, there is a notable male predominance in many studies, with females often underrepresented. This gender disparity is a significant limitation, as menstrual cycle-dependent changes in insulin sensitivity can affect glucose management, highlighting the need for more research focused on females to better understand gender-specific responses to exercise and diabetes management.

Nonetheless, this systematic review has several strengths that should be considered. To our knowledge, this is the first systematic review to comprehensively address the available literature on long-distance running in athletes with diabetes. Methodologically, an extensive quality assessment of the included studies was performed. Additionally, two authors blinded to each other independently performed and reviewed the systematic literature search and extracted data to minimize selection bias. This review provides valuable context for further studies and offers recommendations for athletes with diabetes regarding management of glycemic control. Given the relatively high prevalence of diabetes mellitus and the widespread popularity of long-distance exercise, this review has significant implications for athletes, coaches, sports scientists, and sports medicine practitioners.

### Recommendations for Future Research

Noteworthy, there remains a significant gap in the available data on long-distance athletes with diabetes. Future research should explore the distinctions between Type 1 and Type 2 diabetes mellitus in athletes, focusing on factors such as glycemic control, nutritional intake, and insulin management. Additionally, with the rapid advancement of technology in diabetes care and management, it would be of considerable interest to assess the impact of CGM and CSII on runners with diabetes, comparing these modern technologies to traditional glucose measurements and MDI, particularly in competitive settings and daily life. The issue of late-onset hypoglycemia and strategies for its management remains an area in which further research is needed, especially for individuals with low hypoglycemia awareness and high glycemic variability. Furthermore, the increasing use of CGM by non-athletes with diabetes presents an intriguing opportunity to investigate its effects on performance, specifically in optimizing glucose uptake and dynamics during both training and competitive events. Future research focused on gender-specific responses to exercise and diabetes management is crucial to understanding these variations and ensuring that both male and female athletes receive personalized care and guidance.

## Conclusion

Research on long-distance runners with diabetes remains limited, with most studies emerging in the past decade. However, evidence suggests that with proper preparation, athletes with diabetes can safely participate in endurance events, including ultra-marathons. This review highlights that endurance athletes with diabetes generally maintain well-regulated blood glucose levels, with a low incidence of hypoglycemia during races as well as late-onset hypoglycemia. Insulin adjustments, particularly basal reductions, play a crucial role in minimizing exercise-induced hypoglycemia. CGM combined with CSII, ideally in the form of AID systems, provide an effective strategy for optimal glucose management, helping athletes to avoid glucose fluctuations and preventing dysglycemia. Additionally, CGM may offer performance benefits beyond diabetes management, potentially aiding endurance athletes without diabetes. Endurance exercise induces significant metabolic and biochemical adaptations in athletes with diabetes, emphasizing the need for further research in this area. Notably, there is a significant gap in research concerning athletes with Type 2 diabetes and female athletes. Given the importance of physical activity in diabetes management, larger trials are essential to determine optimal glycemic control strategies for athletes with diabetes. To maximize safety and performance, athletes with diabetes should engage in shared decision-making with sports physicians, endocrinologists, and nutritionists to tailor individualized diabetes management strategies during training and competition.

## Data Availability

This systematic review is based exclusively on data reported in previously published studies, all of which are fully cited within the reference list of this manuscript. No novel datasets were generated during the current study. All data underlying the analyses and conclusions presented here are available within the corresponding publications cited throughout this review and can be accessed via the respective journals or databases in which they were originally published.

## Abbreviations

ACTH: Adrenocorticotropic hormone
AI: Artificial intelligence
AID: Automated insulin delivery
CGM: Continuous glucose monitoring
CK: Creatine kinase
CSII: Continuous subcutaneous insulin infusion
GH: Growth hormone
GLUT-4: Glucose transporter type 4
HbA_1c_: Glycated hemoglobin
MDI: Multiple daily injection
MeSH: Medical subject headings
PRISMA: Preferred Reporting Items for Systematic Reviews and Meta-Analyses
TAR: Time-above-range
TBR: Time-below-range
TIR: Time-in-range
t-PA: Tissue-type plasminogen activator
u-PA: Urokinase-type plasminogen activator
